# Violence Against Parents by Adult Children: A Systematic Literature Review of Empirical Studies

**DOI:** 10.1177/15248380241280955

**Published:** 2024-09-25

**Authors:** Monika Klun, Aleš Bučar Ručman, Danijela Frangež

**Affiliations:** 1 University of Maribor, Faculty of Criminal Justice and Security, Slovenia

**Keywords:** violence, parent, adult child, violence against parents by adult children, elder abuse

## Abstract

Violence against parents by adult children is the abuse of physical, psychological, social, or economic power committed by a grown-up child against his/her parent or foster parent with the intention of achieving a specific goal such as control, subordination, economic gains, or internal satisfaction. It encompasses physical, psychological, sexual, and financial violence, neglect, and property crime. This paper aims to systematically review the literature on violence against parents by adult children. With the use of a range of keywords, the databases Web of Science, SAGE, SpringerLink, Taylor and Francis online, PubMed, EBSCOhost, JSTOR, ProQuest Ebook Central, PsycInfo, Routledge, and Science Direct were reviewed according to the PRISMA Statement. A review of the literature by key authors and contributions using the snowball method followed. The inclusion criteria were empirical journal papers and books published in English between 1990 and 2023 that address violence against parents by adult children. Exclusion criteria included reviews, commentaries, abstracts, and any publications that address the subject topic in a language other than English. The searches returned 39 relevant contributions. Four common themes emerged across the field: the prevalence and characteristics of violence against parents by adult children, parents’ responses and experiences of violence by adult children, social perceptions of (older) parent abuse, and consequences and prevention of violence against parents by adult children. Findings of the literature review show that violence against parents is an under-researched phenomenon and points to the need for further research, including the prevention, detection, consequences, and treatment of such violence.

## Introduction

Violence against parents by children was first distinguished from other types of family violence by Harbin and Madden (1979) who described it as “battered parent syndrome.” This referred to “actual physical assaults or verbal and nonverbal threats of physical harm” against parents by adolescent and adult children (Harbin & Madden, 1979, p. 1288). Adolescence is the period between childhood and adulthood, from 10 to 19 years of age (World Health Organization, n.d.), noting that the minimum age of criminal responsibility varies around the world. The Committee on the Rights of the Child (2007) recommends setting the minimum age of criminal responsibility at 12 years of age, with 18 years as the upper age limit for juvenile justice. Still, some states also consider individuals older than 18, usually up until age 21, as juveniles. According to Bacalso and Mihajlović (2018, p. 9), “the global average age of criminal responsibility is 12.1 years.” In this paper, we discuss violence against parents by their adult children. Adulthood is typically recognized as the age of legal majority, namely, 18 years in over half the countries across the globe (Bacalso & Mihajlović, 2018). This age of legal majority also applies in most countries where the research presented in this paper was conducted, such as European Member States (European Union Agency for Fundamental Rights, n.d.), Australia, Israel, Norway, Russia, Taiwan, Turkey, and the majority of the United States. In the Republic of Korea, the age of majority is 19 years, while in Japan, it is 20 years (World Population Review, n.d.). However, an evident issue in most of the analyzed research is the lack of a clear definition of adult child and the age range being considered included in the study. The age of adult children is specified in three research studies as 18 years and above (Hamilton & Harris, 2023; Johnson et al., 2018, 2022; Klun et al., 2022). Alongside defining 18 years or more, Anetzberger (1986) and Anetzberger et al. (1994) also indicated the gender of the adult child (son or daughter).

Violence against parents by adult children coincides with elder abuse when the victims in the parent–children relationship are elderly (Holt & Shon, 2018; Klun & Frangež, 2019) (i.e., 60 or 65 years and older (Benbow et al., 2019)), with some research (e.g., Blakely & Morris, 1992; Naughton et al., 2012; Powell & Berman, 2006; Storey & Perka, 2018) showing that the most common perpetrators of elder abuse are the victim’s adult children. In violence against parents, terms such as child-to-parent violence, adolescent-to-parent abuse, and youth aggression in the home usually refer to violence against parents perpetrated by adolescents (Holt & Shon, 2018; Toole-Anstey et al., 2023). As a result, adult perpetrators are mainly left out of the literature on violence against parents. This can be ascribed to the dominant focus of criminologists on juvenile delinquency and violence against parents by adolescent children, the specific style of media reports on violence against parents (Holt & Shon, 2018), along with the double or multifaceted stigmatization of parents or the entire family (Klun & Frangež, 2019). The differences between violence caused by adolescent and adult children and elder abuse lie in the perpetrators, victims, and forms of violence. Perpetrators of the first type of violence are adolescents, whereas perpetrators of the second type of violence are adult children. Powell and Berman (2006) show that other relatives (e.g., siblings, grandchildren), partners, or caregivers at home can also be perpetrators of elder abuse. Victims of elder abuse are people older than 60 or 65 (Benbow et al., 2019; Holt & Shon, 2018). Both types of violence include physical, psychological, sexual, and financial violence, and property crime (e.g., thefts) (Band-Winterstein et al., 2015; Cottrell, 2004; Hamilton & Harris, 2023; Johnson et al., 2018). While parents who are victims of violence by adult children and the elderly may be victims of neglect, violence against parents by adolescent children does not include such a form of violence. Parallels between violence committed by adolescence and adult children and elder abuse emerged when elder abuse and violence against parents were recognized as social problems, notably in the latter half of the 20th century (Anetzberger, 2005, Harbin & Madden, 1979). The shared features of elder abuse, violence against parents by adult children and by adolescents also encompass these crimes’ hidden nature and factors that contribute to underreporting (Cottrell & Monk, 2004; Dow et al., 2020; Miles & Condry, 2015; Smith, 2015, 2022; Quinn & Tomita, 1997). While elder abuse and violence against parents by adolescents are not defined uniformly (Anetzberger, 2005; Hunter et al., 2010), there is a general lack of definition(s) in the field of violence against parents by adult children (e.g., Avieli et al., 2015; Daskalopoulos, Kakouros et al., 2006; Daskalopoulos, Mullin et al., 2006; Korbin et al., 1991; Rapoza, 2006; Steinmetz, 1990; von Heydrich et al., 2012).

Violence against parents by adult children is single, repeated, or continuous violent behavior by an adult child that involves physical or psychological harm that causes the parents to feel threatened, intimidated, or controlled (Klun & Frangež, 2019; Klun et al., 2022). Violence includes any behavior by an individual who deliberately threatens or causes physical, sexual, or psychological harm to others or themselves (Stanko, 2001). The perpetrator harms the victim on purpose or knowingly intends to or exposes the victim to something unwanted. Violent crime involves the breaking of rules and deliberate causing of harm (Felson, 2009), including the deprivation or lack of necessities for development (Butchart & Mikton, 2014). We understand violence against parents by adult children as the abuse of power (physical, psychological, social, and economic) committed by a grown-up child against his/her parent or foster parent to achieve a specific goal (control, subordination, economic gains, internal satisfaction, etc.).

Violence against parents by adult children is connected to myths, which influence the parents’ responses to violence committed by their children, their experience and recognition of violence, as well as the broader social perception of this form of violence. Payne (2011) describes the myths that adult children are violent to their parents because they were victims of violence in childhood and that the violence is an outcome of the parent’s dependence on the child. Beliefs that this violence is less severe than violence committed by a non-family member and less acceptable than violence between spouses (Dow et al., 2020; Homer & Gilleard, 1990) can lead to underestimating the prevalence of the problem of violence against parents by adult children and, in turn, to a smaller number of cases being reported by the victims and other people who are familiar with such cases.

We are witnessing global social changes such as higher education levels, rising economic costs and real-estate costs, a weakening of the labor market (e.g., temporary jobs, unemployment), and changes in the establishing of family life (e.g., delayed marriage and childbearing) (Cobb-Clark, 2008; Kirby & Hoang, 2018; Martínez-Granado& Ruiz-Castillo, 2002). This means that children in adulthood often need emotional and financial support from their parents and choose to or are even forced to live together with them. Although close relationships between parents and their children can provide a secure environment for a child’s transition to adulthood, parents’ excessive interference in their children’s lives can strain family relationships. When young adults encounter difficulties in achieving independence due to ambitious expectations and roles assigned to them, they are often treated as children and adults simultaneously. This can cause confusion and conflict, which may lead or be related to violence against parents (Amstadter et al., 2011; Clemens & Axelson, 1985; Harbin & Madden, 1979; Kirby & Hoang, 2018; Pillemer, 1985; White, 2002). Further, an aging population is emerging, leading to expectations of increased victimization among older people (Podnieks & Thomas, 2017; Yan et al., 2015). Such victimization may be related to the caregiving that older adults require in their advanced age. Along with the rise in life expectancy, there will be a greater need to provide care for older adults in both institutional and domestic settings. It is also worth noting that women, who are typically the primary caregivers (Steinmetz, 1990), tend to live longer than men, with an average gap of 7 years in survival rates for older adults, which “creates a demographic context in which caregiving and the risk of elder abuse shifts from the spouse of the older adult to the adult children” (Schiamberg & Gans, 2000, p. 330). One may thus conclude that the topic of violence against parents by adult children will become even more relevant in the near future and must be further studied. The aim of this paper is to present a systematic literature review of empirical studies on violence against parents by adult children. We address four research questions: (a) What is the prevalence and characteristics of violence against parents caused by adult children? (b) How do the parents experience violence against them? (c) How does society perceive such violence? (d) What are the consequences and possibilities for preventing violence against parents by adult children? These questions highlight the importance of recognizing and detecting this type of violence. Investigation of these issues is relevant for policymakers and practitioners working in police, health, welfare, and other domestic abuse services as well as for researchers in applied and theoretical fields. Literature on violence against parents by adult children is reviewed to answer these questions.

## Methods

**Figure 1.**
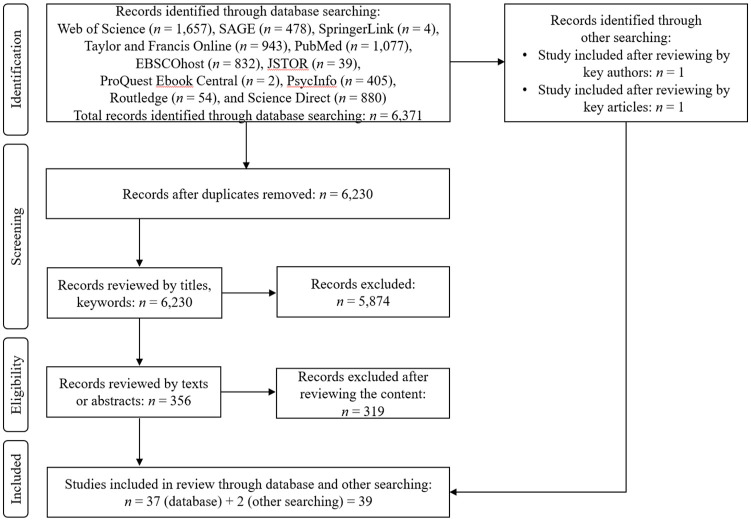
Flow diagram of the systematic literature review.

Our research included a systematic literature review of empirical studies on violence against parents by their adult children. Literature in the databases Web of Science, SAGE, SpringerLink, Taylor and Francis Online, PubMed, EBSCOhost, JSTOR, ProQuest Ebook Central, PsycInfo, Routledge, and Science Direct was reviewed using the PRISMA Statement (Preferred Reporting Items for Systematic reviews and Meta-Analyses), as described in Moher et al. (2009). Conducted between July 2022 to April 2023, the search used the following terms: “elder* AND abus* AND child*” (searched in the title, keyword/subject fields), “elder* AND abus* AND child*” (searched in the content/abstract/text/series title and keyword/subject fields), “violence AND parents AND child*” (searched in the title and keyword/subject fields), “violen* AND parent* AND child*” (searched in the title and keyword fields), “violence against parents” (searched in the title and keyword/subject fields), “elder* AND abus* AND adult* AND child*” (searched in the keyword/subject fields). The inclusion criteria were journal empirical papers and books addressing violence against parents by adult children published in English between 1990 and 2023. The exclusion criteria were reviews, commentaries, abstracts, and any publications that address the subject topic in a language other than English. In the literature review procedure, we initially considered 6,371 records identified by searching the database, before removing duplicates (141). The first author of this paper then reviewed 6,230 records by their titles and keywords and excluded 5,874 records consisting of commentaries, abstracts, and publications in languages other than English. This meant 356 records of empirical studies written in English relevant to the analyzed topic underwent a more detailed review by looking at their texts or abstracts. In the next step, 319 records that did not comprise empirical research on violence against parents by adult children were excluded. In the in-depth analysis (which included full-text screening), 37 records of empirical research on violence against parents by adult children were identified in the database search. Key authors along with papers written by them were identified in the following step. Based on these papers, a literature review was performed using the snowball method according to Wohlin (2014). Two additional references were found and included in the final analysis (Supplemental material Appendix A), which considered a total of 39. Details of the literature review selection process are shown in Figure 1.

The procedure of reviewing the records was documented in Word and Excel files using Microsoft 365 Apps for Business developed by Microsoft (Microsoft Corporation, Redmond, Washington, United States). The Word documents were structured in chapters, each labeled with a keyword, documenting the review procedure. The Excel files were arranged into sheets named after keywords and contained detailed data on the empirical studies selected for the final analysis. The literature was reviewed by the first author of this paper. In the analysis, all research, including nonrepresentative (87.2%) and representative (5.1%) samples, was included, noting that 7.7% of the research did not include information about the sampling. The results of the systematic literature review of empirical studies addressed our research questions using the PEO framework (Bettany-Saltikov, 2012). For the letter P as a population and its problems, we determined the phrase “parents who are victims of violence by adult children” and the word “society,” for E exposure to the term “violence by adult children,” and O as outcomes or themes the words “prevalence,” “characteristics,” “experience,” “perceive,” “consequences,” and the phrase “possibilities for prevention.” Building on this, four critical themes in the literature were revealed: (a) the prevalence and characteristics of violence against parents by adult children; (b) parents’ responses to and experiences of violence by adult children; (c) social perceptions of (older) parent abuse; and (d) consequences and prevention of violence against parents by adult children. These themes are discussed below.

## Results

### Study Characteristics

The literature search yielded 39 studies. The review was international and included research conducted in the United States (*n* = 13), Israel (*n* = 6), Japan (*n* = 5), Australia (*n* = 2), Sweden (*n* = 2), Taiwan (*n* = 2), Germany (*n* = 1), Greece (*n* = 1), Italia (*n* = 1), Korea (*n* = 1), Norway (*n* = 1), Russia (*n* = 1), Slovenia (*n* = 1), Turkey (*n* = 1), and United Kingdom (*n* = 1). Among these, 14 studies were quantitative and 25 were qualitative. Most studies examined violence against parents by adult children from sociological perspectives related to the prevalence and characteristics (*n* = 16), while a few addressed social perceptions of (older) parent abuse (*n* = 8). Six studies examined the issues of parents’ responses to and experiences of violence by adult children, and five studies were concerned with the consequences and prevention of violence against parents by adult children. Three studies examined two themes (prevalence and characteristics/parents’ responses to and experiences of violence by adult children; prevalence and characteristics/consequences and prevention; parents’ responses to and experiences of violence by adult children/consequences and prevention), while one study was related to three themes (prevalence and characteristics/parents’ responses to and experiences of violence by adult children/consequences and prevention). Quantitative research typically deals with the prevalence and characteristics, while qualitative research deals with the perception of parents and society. The analysis of the contributions is summarized in Table 1.

**Table 1.** Details of Empirical Studies on Violence Against Parents by Adult Children Included in the Review.

Author(s), Year of Publication; Country	Type of Research; Study Design, Data Collection	Participants (Age; Sample Size); Sampling	Type of Violence	Main Findings
1. Aas (2018); Norway	Qualitative study; exploratory study, semi-structured interviews	Police officers, employees of the Elderly Protection Service in Norway, and managers of four shelters (no data; *n* = 27); purposive	Not specified	Police officers must be flexible and face some dilemmas while dealing the cases of violence against parents by adult children
2. Anetzberger (1986); United States	Qualitative study; exploratory study, (semi)structured interviews	Adult children (mostly 30–60 years; *n* = 15); purposive	Physical	Types of perpetrators: hostile, authoritarian, dependent
3. Anetzberger et al. (1994); United States	Qualitative study; exploratory study, (semi)structured interviews	Adult children (*M* = 51.5 years; *n* = 62); not reported	Physical	Perpetrators are often unmarried and more likely than non-abusive children to be identified as alcoholics
4. Avieli et al. (2015); Israel	Qualitative study; phenomenological study, semi-structured interviews	Parents (58–94 years; *n* = 20); purposive	Psychological, physical, financial	Types of departure scripts: a pragmatic farewell script, a burned-out farewell script, a dead-end farewell script, an optimistic farewell script, and a violent farewell script
5. Band-Winterstein et al. (2014); Israel	Qualitative study; phenomenological study, semi-structured interviews	Parents (58–94 years; *n* = 16); purposive	Psychological, physical, financial	The challenges and complexities faced by aging parents who are caring for children with mental disorders can be grouped in themes: age as a platform for parental vulnerability in the face of ongoing abuse; the shared reality of abusive and vulnerable protagonists; and changes in relationship dynamics
6. Band-Winterstein (2015); Israel	Qualitative study; phenomenological study, semi-structured interviews	Parents (65–91 years; *n* = 12) and children (30–50 years; *n* = 11); purposive	Psychological, physical, financial, neglect	Types of victimhood: the elderly parent as the central victim of suffering and the perpetrator as the secondary victim; the perpetrator as the primary victim of suffering and the parent as both victimizer and victim; the perpetrator as martyr vs. the parent as a martyr; a reciprocal narrative of suffering
7. Band-Winterstein et al. (2015); Israel	Qualitative study; phenomenological study, semi-structured interviews	Parents (58–94 years; *n* = 16); purposive	Psychological, physical, financial	Constructing the parental identity (devoted, “martyrdom,” bitter and deprived, brave)
8. Band-Winterstein et al. (2016); Israel	Qualitative study; phenomenological study, semi-structured interviews	Parents (58–94 years; *n* = 16); purposive	Psychological, physical, financial	Parents experience the child’s behavior along two continuums (more harmed vs. more harmful, normative vs. pathological). A typology of parental perceptions and definitions of children’s deviant behaviors: a harmed-pathological behavior, a harmful-pathological behavior, a harmed-normative behavior, a harmful-normative behavior, and a diffused perception.
9. Daskalopoulos and Borrelli (2006); Italia	Open-ended qualitative survey; exploratory study forming part of a larger study “Cross-Cultural Definitions of Family Violence and Abuse Survey” or part of the project “Cross-Cultural Perspectives on Violence and Abuse Research Project” at Boston University (Malley-Morrison, 2004, 2006)	Community sample (21–48 years; *n* = 53); convenience sampling	Not specified	Most examples of psychological abuse of adult children toward parents and neglect provided by respondents from the community sample were prevalent in examples of moderate and mild abuse toward the elderly person
10. Daskalopoulos, Kakouros et al. (2006); Greece	Open-ended qualitative survey; exploratory study forming part of a larger study “Cross-Cultural Definitions of Family Violence and Abuse Survey” or part of the project “Cross-Cultural Perspectives on Violence and Abuse Research Project” at Boston University (Malley-Morrison, 2004, 2006)	Community sample (17–87 years; *n* = 71); convenience sampling	Not specified	Respondents from a community sample often provided examples of psychological abuse by adult children toward parents and neglect as common forms of moderate abuse toward the elderly person. On the mild level, the most frequent form of abuse was neglect of the parents.
11. Daskalopoulos, Mullin et al. (2006); United Kingdom	Open-ended qualitative survey; exploratory study forming part of a larger study “Cross-Cultural Definitions of Family Violence and Abuse Survey” or part of the project “Cross-Cultural Perspectives on Violence and Abuse Research Project” at Boston University (Malley-Morrison, 2004, 2006)	Community sample (20–73 years; *n* = 50); convenience sampling	Not specified	The forms of neglect and psychological abuse of adult children toward parents stated by respondents from a community sample were the most common examples of moderate and mild abuse toward the elderly person. Society is resistant to violence against parents by adult children.
12. Dong and Li (2015); United States	Quantitative study; cross-sectional survey, self-report questionnaire	Adult children (22–75 years; *n* = 548); purposive	Psychological, financial	59.8% of adult children disclosed the risk of violence against their parents
13. Dong et al. (2017); United States	Quantitative study; cross-sectional survey, self-report questionnaire	Adult children (22–75 years; *n* = 548); purposive	Psychological, financial	Risk factor for parents: childhood maltreatment
14. Dow et al. (2020); Australia	Qualitative study; exploratory study, semi-structured interviews	Elderly parents (62–89 years; *n* = 28); purposive	Not specified	Reasons for nonreporting: parental bond, love, responsibility, shame, fear
15. Golding et al. (2013); United States	Quantitative study; experiment, mock juror paradigm	Undergraduate psychology students (18–21 years; *n* = 143) and community sample (18–81 years; *n* = 104); not reported	Financial	Respondents as mock jurors were given a scenario involving the financial exploitation of a mother by her adult son, with two variables being manipulated (the victim’s medical condition and the amount of money). When the victim was described as healthy, the mock jurors were more likely to render guilty verdicts than when the alleged victim was described as having cognitive problems. The amount of alleged stolen money predicted guilty verdicts.
16. Greenberg et al. (1990); United States	Qualitative study; exploratory study analyzing cases of violence against parents by an adult child	Cases of violence against parents by adult children (*M* (victims) = 76.5, years; *M* (children) = 43 years; *n* = 204); purposive	Physical, financial, neglect	Victims are often female, on average 76.5 years old. 32% of sons and 25% of daughters were unemployed; perpetrators can be financially dependent and dependent due to problems related to mental illness
17. Hamilton and Harris (2023); Australia	Qualitative study; exploratory study, case studies	Cases of violence against parents by adult children (children, parents: no data about age; *n* = 11); collective case study approach	Not specified	Characteristics of perpetrators: depression, mental health problems, alcohol and other drug problems, and unemployment
18. Hsu and Tu (2014); Taiwan	Qualitative study; phenomenological study, semi-structured interviews	Adult children (*M* = 35.6 years; *n* = 14); purposive	Not specified	Violence occurring in repeated episodes that cause physical and psychological consequences for the parents
19. Johnson et al. (2018); Sweden	Quantitative study; cross-sectional survey, self-report questionnaire	Parents (mostly 50–59 years; *n* = 687); purposive	Property crime	Over half the parents reported that their children had stolen something from them
20. Johnson et al. (2022); Sweden	Quantitative study; exploratory study, (self-report) questionnaire	Parents (35–84 years; *n* = 687); purposive	Physical violence, property crime	19% of parents had been victims of physical violence at any time in their lives, and 6.2% in 2014. 40% of parents had been victims of property crime at any time in their lives, and 10% in 2014
21. Kageyama and Solomon (2018)^1^; Japan	Quantitative study; cross-sectional study, questionnaire	Parents (mostly 60–69 years; *n* = 353); purposive	Psychological, physical	Parents who experience violence by their adult child with schizophrenia have a risk of developing post-traumatic stress syndrome
22. Kageyama, Solomon, Kita, et al. (2016); Japan	Quantitative study; cross-sectional survey, self-report questionnaire	Parents (no data about age; *n* = 400); purposive	Physical	One-third of parents reported physical violence by adult children with schizophrenia
23. Kageyama, Solomon, Yokoyama (2016); Japan	Quantitative study; cross-sectional survey, self-report questionnaire	Parents (*M* = 69 years; *n* = 379); purposive	Psychological, physical	34.8% of parents reported having experienced physical and 25.3% psychological violence by their children with schizophrenia in the previous year
24. Kageyama et al. (2018); Japan	Qualitative study; phenomenological study, semi-structured interviews	Parents (50–83 years; *n* = 26); snowball	Physical	A four-stage coping process of parent victims of violence caused by adult children with schizophrenia was identified: hope for treatment, living with violence, attempting to resolve the violence, and final resolution to the violence
25. Kageyama et al. (2020); Japan	Quantitative study; experiment	Parents (*M* = 68.6; *n* = 66); purposive	Psychological, physical, financial, sexual	The video-based educational program is a potentially successful tool for family violence prevention and communication between parents and their adult schizophrenia children
26. Klun et al. (2022); Slovenia	Quantitative study; retrospective study, analysis of police statistics	Police statistics; purposive	Manslaughter, murder, actual bodily harm, aggravated bodily harm, grievous bodily harm, sexual abuse of a defenseless person, and family violence	The share of cases of violence against parents concerning all crimes ranged between 4% and 7% per year
27. König and Leembruggen-Kallberg (2006); Germany	Open-ended qualitative survey; exploratory study forming part of a larger study “Cross-Cultural Definitions of Family Violence and Abuse Survey” or part of the project “Cross-Cultural Perspectives on Violence and Abuse Research Project” at Boston University (Malley-Morrison, 2004, 2006)	Community sample (15–62 years; *n* = 74); convenience sampling	Not specified	Respondents from a community sample more frequently cited examples of psychological abuse of parents by adult children in cases of moderate elder abuse rather than extreme elder abuse. Men described more physical aggression examples than women when providing examples of moderate elder abuse.
28. Korbin et al. (1991); United States	Qualitative study; exploratory study, (semi)structured interviews	Elderly parents (over 60 years; *n* = 29); purposive	Physical, sexual, financial	Victims’ average age is 72.1 years. Characteristics of perpetrators: usually sons, mean age: 44.1 years, mental illness, substance abuse
29. Korbin et al. (1995); United States	Quantitative study; cross-sectional survey, self-report questionnaire	Adult children (*M* = 48 years; *n* = 23) and elderly parents (no data about age; *n* = 21); purposive	Physical	Parents who abused their children were more likely to report more serious forms of violent behavior in their own childhood compared to adult offspring who abused their elderly parents
30. Lee et al. (2022)^2^; Korea	Quantitative study; exploratory study, (self-report) questionnaire	Adult children (*M* = 39.9 years; *n* = 2,974); randomly	Psychological	The prevalence of psychological violence is 8.7%
31. Marcus et al. (2005); Israel	Qualitative study; exploratory study, case study	Case study of violence against mother (*n* = 1); purposive	Neglect	Providing a vegan diet to the mother was considered to be violence (physical neglect of an older adult due to negligence)
32. Rapoza (2006); United States	Open-ended qualitative survey; exploratory study forming part of a larger study “Cross-Cultural Definitions of Family Violence and Abuse Survey” or part of the project “Cross-Cultural Perspectives on Violence and Abuse Research Project” at Boston University (Malley-Morrison, 2004, 2006)	Community sample (17–79 years; *n* = 76); convenience sampling	Not specified	Community sample respondents’ examples of psychological abuse of parents by adult children were the most common examples of moderate abuse toward the elderly person. There was a statistically significant relationship between age and cases of verbal abuse of adult children toward parents at the mild level of abuse toward an elderly person.
33. Rinsky and Malley-Morrison (2006); Russia	Open-ended qualitative survey; exploratory study forming part of a larger study “Cross-Cultural Definitions of Family Violence and Abuse Survey” or part of the project “Cross-Cultural Perspectives on Violence and Abuse Research Project” at Boston University (Malley-Morrison, 2004, 2006)	Community sample (17–43 years; *n* = 21); convenience sampling	Not specified	The most frequent examples of moderate abuse toward the elderly person provided by community sample respondents were psychological abuse and the neglect of parents by adult children
34. Smith (2015); United States	Qualitative study; exploratory study, semi-structured interviews	Elderly parents (over 62 years; *n* = 15); purposive	Psychological, physical	Mothers experienced various pressures when the child returned home, for example, children violated boundaries and there was a lack of reciprocity between mothers and children. Mothers had internal conflicts due to (not) responding to the pressures they felt when living with the child.
35. Smith (2022); United States	Qualitative study; exploratory study, semi-structured interviews	Elderly parents (over 60 years; *n* = 29); not reported	Psychological, physical, financial	Mothers face conflict between protecting their children and themselves, feeling love and hate toward their adult children. They are conflicted about removing their child or calling the police.
36. Steinmetz (1990); United States	Qualitative study; exploratory study, (semi)structured interviews	Adult children (mostly 50–59 years; *n* = 104); snowball	Physical, psychological	The perception of stress due to caregiving may be related more to the likelihood of violence than the actual amount of care provided
37. Sun and Hsu (2016); Taiwan	Quantitative study; experiment	Adult children (20 years and above) and parents (no data about age; 36 dyads in experimental groups and 33 dyads in the control group); purposive	Psychological, physical)	The Child- and Parent-focused Violence Program is an effective preventive program
38. von Heydrich et al. (2012)^3^; United States	Quantitative study; longitudinal study, phone and self-administered questionnaires	Elderly parents (65 years and above; *n* = 203); randomly	Physical, sexual	5.9% of elderly parents reported having been physically abused, and 9.4% indicated they had been sexually abused
39. Yalçinkaya et al. (2006); Turkey	Open-ended qualitative survey; exploratory study forming part of a larger study “Cross-Cultural Definitions of Family Violence and Abuse Survey” or part of the project “Cross-Cultural Perspectives on Violence and Abuse Research Project” at Boston University (Malley-Morrison, 2004, 2006)	Community sample (22–58 years; *n* = 39); convenience sampling	Not specified	The most common examples of moderate and mild abuse toward the older person provided by the respondents from a community sample were examples of psychological abuse of adult children toward parents. Examples of physical abuse toward parents were given only as extreme abuse.

### Prevalence and Characteristics of Violence Against Parents by Adult Children

Estimating the prevalence of violence against parents by adult children is a challenging endeavor. Dow et al. (2020) found that the parental bond prevents the reporting of violence. Parents feel a strong love, responsibility, and obligation to protect their children despite their violent behavior. Factors in nonreporting can also be experiencing feelings of shame, fear of negative consequences for the perpetrator and themselves (e.g., a fear of retaliation by the child, a fear of losing contact with the grandchildren), and feelings of guilt.

Research findings show that the prevalence of violence against parents ranges from 5.9% to 40% (Dong & Li, 2015; Johnson et al., 2018, 2022; Kageyama, Solomon, Yokoyama, 2016; Klun et al., 2022; Lee et al., 2022; von Heydrich et al., 2012). Von Heydrich et al. (2012) established that 5.9% of elderly parents reported having been physically abused. A survey in Japan (Kageyama, Solomon, Kita, et al., 2016) revealed that one-third of parents had experienced physical violence by their adult children with schizophrenia in the past year. Similarly, Kageyama, Solomon, Yokoyama (2016) determined that 34.8% of parents reported having experienced physical violence by their children with schizophrenia in the previous year. Johnson et al. (2022) found that in Sweden 19% of parents had been victims of physical violence at any time in their lives, and 6.2% in 2014. Using the Caregiver Abuse Screen, Dong and Li (2015) established that almost 60% of adult children among the United States Chinese population revealed the risk of (psychological and/or financial) violence being perpetrated against their elderly parents. Lee et al. (2022) found that the prevalence of psychological violence in Korea is 8.7%. A survey by Kageyama, Solomon, Yokoyama (2016) showed that 25.3% of parents reported psychological violence by their children with schizophrenia in the previous year.

The prevalence of property crimes is slightly higher, with Johnson et al. (2018) finding that in Sweden more than half the parents reported that their children had stolen something from them. A few years later, Johnson et al. (2022) established that 40% of parents had been victims of property crime at any time in their lives, and 10% in 2014.

Von Heydrich et al. (2012) revealed 9.4% of parents had been sexually abused. Between 2010 and 2020, the Slovenian police dealt with two cases of sexual abuse of a defenseless person in an adult child–parent relationship. These cases, along with cases of grievous bodily harm, represented the lowest number of crimes in the adult child–parent relationships investigated by the Slovenian police. The most common crime was family violence (11%), followed by actual and aggravated bodily harm. The share of cases of violence against parents concerning all crimes (excluding the sexual abuse of a defenseless person) ranged between 4% and 7% per year (Klun et al., 2022).

Research findings indicate that victims are mostly female. Although studies by Greenberg et al. (1990) and Korbin et al. (1991) included both females and males, only the shares of females were presented in the research, ranging from 74.1% to 77.3%. Kageyama, Solomon, Yokoyama (2016) established that 67.8% of victims were mothers and 32.2% were fathers. Similarly, Kageyama, Solomon, Kita et al. (2016) found that 63% of victims were mothers, while 31.8% were fathers. In terms of victims’ age, Greenberg et al. (1990), Kageyama et al. (2018), and Korbin et al. (1991) determined the average age of female and male victims together. Greenberg et al. (1990) established an average age of 76.5 years, Korbin et al. (1991) an average age of 72.1 years, whereas Kageyama et al. (2018) an average age of 70.8 years. The sample of Korbin et al. (1991) consisted of victims who were over 60 years old, whereas Kageyama et al. (2018) analyzed victims who were between 50 and 83 years old.

Perpetrators are generally men (Korbin et al., 1991), on average aged 39.6 to 44.1 years (Greenberg et al., 1990; Kageyama et al., 2018; Korbin et al., 1991). The sample of Kageyama et al. (2018) comprised perpetrators between 20 and 50 years of age. While Korbin et al. (1991) stated the age range (28–57 years) only for perpetrators in cases where parents sought legal recourse due to violence. Anetzberger et al. (1994) found that 65.2% perpetrators had completed high school, and 78.3% were unmarried. According to Greenberg et al. (1990), 32% of male perpetrators and 25% of female ones were unemployed. Perpetrators may have poor health, emotional, or psychological problems (e.g., depression), an attention or autistic disorder (Hamilton & Harris, 2023; Johnson et al., 2018, 2022; Korbin et al., 1991; Lee et al., 2022; von Heydrich et al., 2012), or schizophrenia (Hsu & Tu, 2014; Kageyama, Solomon, Kita et al., 2016; Kageyama, Solomon, Yokoyama, 2016; Kageyama et al., 2018). Some perpetrators misuse alcohol (Hamilton & Harris, 2023; Korbin et al., 1991), which can also be associated with violence against parents (Anetzberger et al., 1994; Johnson et al., 2018; von Heydrich et al., 2012).

Johnson et al. (2022) found that parents of children with drug problems experienced higher levels of physical violence and property damage in the past year. In more detail, parents of children who engaged in cannabis abuse often experience lifetime exposure to physical violence, while new psychoactive substances are associated with lifetime exposure to property damage. Johnson et al. (2018) state that it is challenging to fund a high level of illegal drug consumption on a legal income, leading to increased theft and property offenses against parents by adult children with serious drug problems. Von Heydrich et al. (2012) similarly conclude that substance abuse can cause children financial problems, which can lead to the parents being financially exploited.

Von Heydrich et al. (2012) explain that the child’s financial problems worsen the quality of the relationship with the parent and add to the likelihood of physical abuse. Lee et al. (2022) discovered that married children who commit psychological violence against their parents earn less on average than those who are not violent and that temporarily employed children are more likely to be violent than full-time ones. Further, married daughters were 122.9% more likely to commit psychological violence against their parents than married sons, explained by daughters having an essential role in supporting parents.

Parents and children are more likely to disclose violence if the children are younger and have a higher level of education (Dong & Li, 2015; Johnson et al., 2022). According to von Heydrich et al. (2012), predictors of physical violence against parents are social isolation and parental health. Poorer quality parent–child relationships can reinforce the parent’s social isolation, and vice versa. Parental health relates to caring for the parent, which can be stressful for the child (von Heydrich et al., 2012) and increases possibilities for conflicts. Steinmetz (1990) noted that the likelihood of violence may be related more to the perceived stress of caregiving than the actual amount of care provided. Another characteristic is the perpetrator’s co-residence with their parents. Living with the parents represents a risk factor for violent behavior (Johnson et al., 2022; Lee et al., 2022), which Lee et al. (2022) explain by the fact that conflicts are more likely to occur while cohabitating.

With respect to the model of the intergenerational transmission of violence, on the one hand Korbin et al. (1995) determined that it is more helpful for explaining child abuse than violence against parents by adult children. On the other hand, a more recent study on a sample of Chinese children in the United States supports the model of the intergenerational transmission of violence by showing that child abuse is statistically significantly associated with violence against parents (Dong et al., 2017).

Anetzberger (1986) divided perpetrators who are violent toward their parents into three types: hostiles, authoritarians, and dependents. Hostile perpetrators usually have long-term problems in their relationships with their parents. They believe their parents have psychopathological problems and perceive the caring for their parents as very burdensome. Authoritarian perpetrators are married, have a need for control, and are not mentally ill. They think that their parents were overbearing toward them while they were children. Dependent perpetrators rely financially on their parents and often live with them. They are immature, unmarried, less educated, and unemployed. Greenberg et al. (1990) divided the dependents into perpetrators who have problems related to alcohol and drug abuse and are financially dependent on parents and perpetrators who are mentally ill and mentally reliant on parents.

### Parents’ Responses to and Experiences of Violence by Adult Children

Smith (2015, 2022) found that mothers who were victims of violence by adult children disclosed the various pressures that arose when the child moved back home. In such cases, boundary violations were present, for example, the disobedience of mothers. In addition, there was a lack of reciprocity between mothers and children. Mothers expected that their children would treat them with respect and that the children would become independent, but the children did not meet their expectations. Mothers were often in situations that led to internal conflict. If they acted upon their children’s violence (e.g., reported the violence to the police), they became concerned about their children’s future and safety. They typically did not respond to the children’s challenging behavior because they did not want to be heartless or still hoped the child would change. Further, they experienced a sense of shame, blamed themselves, and attributed violence to their bad parenting.

Moreover, Band-Winterstein et al. (2014) found that parents perceive old age as a platform for their vulnerability facing ongoing abuse. Due to the accumulation of physical and emotional child outbursts, parents feel exhausted and ill, while social bonds weaken with age, leading to feelings of isolation, vulnerability, and powerlessness. Like in Smith (2015, 2022), the conflict over whose needs to come first (their own or their child’s) was also disclosed.

Based on interviews with parents and their abusive adult children, Band-Winterstein (2015) identified four types of victimhood. The first type refers to the parent as a central, primary victim, while the adult child is the secondary or additional victim of this relationship. In this type, parents describe suffering as a significant entity in their lives and the whole family’s lives, and the child’s suffering explains their behavior. The second type of victimhood interprets the adult child as the central, primary victim and the parent as both perpetrator and victim. The third type refers to adult children and parents being perceived as martyrs, while the last type emphasizes the suffering they experience to the same extent.

One study (Band-Winterstein et al., 2015) showed that parents who are victims of a child’s violence form unique groups of identities, regardless of whether the violence is caused by a mental disorder of the child or other factors. Based on interviews, one set of parents perceived their parenthood as devoted; another group as “martyrdom,” another group perceived it as bitter and deprived parenthood, while another as bravery against all difficulties. Moreover, Band-Winterstein et al. (2016) determined that parents of violent children with mental disorders perceive themselves as responsible for the family and supporting the adult child. Parents experience a child’s deviant behavior along two continuums. The first represents the child’s perception as more harmed versus more harmful, while the second represents the child’s perception as normative versus pathological. Based on these continuums, a typology of parental perceptions and definitions of children’s deviant behaviors have formed groups of behaviors with matching experiences of parents’ feelings: harmed-pathological behavior, where parents have emotions based on love- commitment-perseverance; harmful-pathological behavior, marked by parents’ emotions based on a lack of love-helplessness-misery; harmed-normative behavior, characterized by parents’ emotions based on overprotection-controlling through instructions-infantile feelings; and harmful-normative behavior, where parents feel ambivalent emotions based on closeness–distance.

Parents felt burdened and frustrated because they could not deal effectively with their child’s violent behavior (Hsu & Tu, 2014). Further, parents expressed a desire to control the violent behavior and agreed that managing the violence was possible. Kageyama et al. (2018) concluded that the coping process of parent victims of violence caused by children with schizophrenia undergoes different stages. In the first stage (Hope for treatment), when the violence began, the parents saw the illness as the problem, not the violence. Parents sought treatment in most cases, although this did not help to stop the violence. In the second stage (Living with violence), they tried to understand the characteristics and causes of the violence. They did not talk about the violence due to feelings of shame, love, and responsibility and worked to change themselves. In the third stage (Trying to solve violence), they sought help and support, and some parents recognized that violence is a severe problem. The fourth stage (Last solution for violence) usually follows when many families break up after a long period of violence. They realized that the last key step was to separate the parents from the children and live independently. Avieli et al. (2015) examined end-of-life preparations among elderly parents of mentally ill, abusive children in the context of life reviews. Various departure scripts regarding how they would ultimately separate from their child emerged: a pragmatic departure script, a burned-out departure script, a dead-end departure script, an optimistic departure script, and a violent departure script.

### Social Perceptions of (Older) Parent Abuse

Studies on how society perceives violence against parents by adult children were conducted in Italy, England, Greece, Russia, Turkey, the United States, and Germany (Daskalopoulos & Borrelli, 2006; Daskalopoulos, Kakouros et al., 2006; Daskalopoulos, Mullin et al., 2006; König & Leembruggen-Kallberg, 2006; Rapoza, 2006; Rinsky & Malley-Morrison, 2006; Yalçinkaya et al., 2006). Respondents were asked to give an example of a situation in which an adult interacted with an older parent whereby most people in their culture would view the adult’s behavior as an extreme, moderate, or mild form of violence. In Italy, there were the most cases of extreme forms of violence related to physical violence and neglect. The most frequent instances of psychological abuse and neglect were moderate and mild. Research conducted in England, Greece, Russia, Turkey, and Germany (Daskalopoulos & Borrelli, 2006; Daskalopoulos, Kakouros et al., 2006; Daskalopoulos, Mullin et al., 2006; König & Leembruggen-Kallberg, 2006; Rapoza, 2006; Rinsky & Malley-Morrison, 2006; Yalçinkaya et al., 2006) show similar findings. In Greece, there is a general opinion that a child must care for their aging dependent parents, with acts of omission being perceived as just as violent (if not more damaging) than acts of commission (Daskalopoulos, Kakouros et al., 2006). In Russia, all participants gave examples of physical violence of hitting the parents and physical violence toward an older father by an adult son (Rinsky & Malley-Morrison, 2006). In Turkey, as an extreme form of violence, only examples of physical abuse were given (Yalçinkaya et al., 2006). In Germany, gender differences were noticed in moderate and mild elder abuse examples, as “men provided more physical aggression response than women when giving examples of moderate abuse” (König & Leembruggen-Kallberg, 2006, p. 32). In the United States, Rapoza (2006) established a statistically significant relationship between age and cases of verbal abuse on the mild level. The older the respondent was, the less they mentioned instances of verbal abuse on the mild level. Rapoza (2006) explains that younger people may have described more of such examples due to social norms, which are still strongly oriented to verbal respect for elders.

People overlook adult children’s violence against their parents when considering the main types of family violence (e.g., child abuse, intimate partner violence) (Daskalopoulos & Borrelli, 2006; Rapoza, 2006), which we can relate to the conclusion drawn by Daskalopoulos, Mullin et al. (2006) that society might be resistant to this type of problem. It is also rare that society would associate the violence of an adult child against a parent with financial exploitation, for example, stealing assets of older parents, taking control of their finances (Daskalopoulos, Mullin et al., 2006; Rapoza, 2006; Rinsky & Malley-Morrison, 2006) and with sexual abuse (Rapoza, 2006; Rinsky & Malley-Morrison, 2006). Rinsky and Malley-Morrison (2006) explain that the latter type of violence may not happen or would never be mentioned if it did happen. Rapoza (2006, p. 30) argues this is because “child-initiated incest is too unthinkable.”

Regarding financial exploitation, Golding et al. (2013) examined the jury’s perception of elder financial exploitation in the courtroom. Mock jurors were presented with a case of financial exploitation of a mother by an adult son, manipulating two variables (the victim’s medical condition—healthy or cognitively impaired and the amount of money: US$ 500 or US$ 5,000). Golding et al. (2013) found that when the description of the alleged victim contained the information that the victim was healthy, respondents were more likely to provide guilty verdicts than when the alleged victim was described as having cognitive problems. The amount of allegedly stolen money was a statistically significant predictor of guilt verdicts. More enormous sums of money allegedly stolen led to a higher rate of guilt verdicts.

### Consequences and Prevention of Violence Against Parents by Adult Children

Only one study that looks at the aspect of the consequences could be found. Kageyama and Solomon (2018) examined the consequences for parents who are victims of violence by their adult children with schizophrenia, and found that experiencing severe forms of violence and the hospitalization of a child are associated with a high risk of post-traumatic stress syndrome in parents. Parents also reported physical injuries (Kageyama et al., 2018) and other medical issues (e.g., high blood pressure, headaches) (Band-Winterstein, 2015). Kageyama, Solomon, Kita et al. (2016) stated that if a violent child is mentally ill, the parents may lose their jobs due to having to stay at home to care for them, further reducing financial resources.

Sun and Hsu (2016) evaluated a parent–adult child intervention program designed to manage child-to-parent violence named the Child- and Parent-focused Violence Program. The study showed that the program is effective for improving the management of violence, alleviating the intensity of the parents’ bio-psychological and emotional reactions, controlling impulsivity, and changing the attitudes of parents and children to violence. Kageyama et al. (2020) conducted a pilot study and assessed a video-based educational program. They found significant improvements in expressed emotion, psychological distress, and family empowerment.

Aas (2018) examined how the police tries to prevent violence caused by adult children against their parents and established that victims usually deny the abuse by their children. At the same time, they are worried about whether the child will receive help because they have, for example, drug abuse problems and psychiatric disorders. After a report is made, to protect the victims, police have the right to prohibit a suspect from being at a certain place or from following, visiting, or contacting the victim (a restraining order). Parents generally do not approve of such an order. They prefer to stay in touch with their children to ensure the safety of their child. Police officers must also be flexible because parents might only have a few years left to live and would be unable to see their child if a restraining order were put in place. Another dilemma is when the victim simply wants to talk but does not wish to formally report the child. Police officers must balance the conflicting interests that arise when, on one side, the elderly person describing their problem does not wish to pursue criminal proceedings and, on the other, the police have a duty to make a report and deal with crimes. In this theme, another form of abuse was related to physical neglect, which resulted in a vitamin deficiency caused by an inappropriate vegan diet (Marcus et al., 2005).

## Discussion

The systematic literature review of empirical studies concerned with violence against parents by adult children shows that the prevalence of violence against parents by their adult children ranges from 5.9% to 40% (Dong & Li, 2015; Johnson et al., 2018, 2022; Kageyama, Solomon, Yokoyama, 2016; Klun et al., 2022; Lee et al., 2022; von Heydrich et al., 2012). The research identified the prevalence of psychological (Kageyama, Solomon, Yokoyama, 2016; Lee et al., 2022), physical (Johnson et al., 2022; Kageyama, Solomon, Kita et al., 2016; Kageyama, Solomon, Yokoyama, 2016; Klun et al., 2022; von Heydrich et al., 2012), and sexual violence (Klun et al., 2022; von Heydrich et al., 2012) as well as property crime (Johnson et al., 2018, 2022).

Kageyama, Solomon, Kita et al. (2016) and Kageyama, Solomon, Yokoyama (2016) concluded that victims are mostly mothers, on average 70.8–76.5 years of age (Greenberg et al., 1990; Kageyama et al., 2018; Korbin et al., 1991). Perpetrators are generally men (Korbin et al., 1991). On average, they are 39.6 to 44.1 years old (Greenberg et al., 1990; Kageyama et al., 2018; Korbin et al., 1991), noting that Anetzberger et al. (1994) established that they are mostly unmarried and have a high school education. It has also been shown that some of them have mental health issues (Hamilton & Harris, 2023; Johnson et al., 2018, 2022; Korbin et al., 1991; Lee et al., 2022; von Heydrich et al., 2012), problems with alcohol and illegal drug abuse (Anetzberger et al., 1994; Hamilton & Harris, 2023; Johnson et al., 2018, 2022; Korbin et al., 1991; von Heydrich et al., 2012), and financial problems (Anetzberger, 1986; Greenberg et al., 1990; Johnson et al., 2022; Lee et al., 2022; von Heydrich et al., 2012). Living in the community with parents has an essential role in violence (Johnson et al., 2022; Lee et al., 2022). Regarding the intergenerational transmission of violence, Korbin et al. (1995) established that the model is better at explaining child abuse, albeit Dong et al. (2017) found a significant association between child abuse and violence against parents.

Research examining how parents experience victimization was mainly conducted in Israel (Avieli et al., 2015; Band-Winterstein, 2015; Band-Winterstein et al., 2014, 2015, 2016). Parents are reluctant to report violence and experience inner frustration because they still love their children. Feelings of shame, fear, and guilt are additional barriers to reporting (Band-Winterstein et al., 2016; Dow et al., 2020; Hsu & Tu, 2014; Smith, 2015, 2022). In a violent relationship, Band-Winterstein (2015) found that different dynamics can develop between a parent and a child whose common thread is shifting the strength and intensity of the violence and the questions of who is the actual perpetrator of the violence and who is the recipient. Band-Winterstein et al. (2014) revealed that the relationship between an abusive child with a mental disorder and the parent is based on mutual dependence, whereas, according to Band-Winterstein et al. (2015), parents perceive parenting through the prisms of devotion, “martyrdom,” deprivation, or courage. A more detailed process of how parents cope with the violence of a child with schizophrenia was investigated by Hsu and Tu (2014), who found that such a relationship consists of four stages, ending with the breakup of the family and the child’s independent life.

Research on the social perception of violence against (older) parents was conducted in the United States (Rapoza, 2006), England (Daskalopoulos, Mullin et al., 2006), Germany (König & Leembruggen-Kallberg, 2006), Greece (Daskalopoulos, Kakouros et al., 2006), Italy (Daskalopoulos & Borrelli, 2006), Russia (Rinsky & Malley-Morrison, 2006), and Turkey (Yalçinkaya et al., 2006). Among the most extreme forms of violence, respondents usually placed physical violence against parents and neglect, while moderate and mild violence included psychological violence (Daskalopoulos, Kakouros et al., 2006; Daskalopoulos, Mullin et al., 2006; König & Leembruggen-Kallberg, 2006; Rapoza, 2006; Rinsky & Malley-Morrison, 2006; Yalçinkaya et al., 2006). Violence against parents may represent resistance in society (Daskalopoulos, Mullin et al., 2006) and such cases are not thought of as quickly as other types of family violence (Daskalopoulos & Borrelli, 2006; Rapoza, 2006).

In terms of consequences, Kageyama and Solomon (2018) found that parents who are victims of violence by children with schizophrenia face a high risk of post-traumatic stress syndrome. Evaluation studies of the Child- and Parent-focused Violence Program (Sun & Hsu, 2016) and a video-based educational program (Kageyama et al., 2020) showed promising results. The study by Sun and Hsu (2016) proved that the Child- and Parent-focused Violence Program was effective for managing violence, alleviating parents’ bio-psychological and emotional reactions, managing impulsivity, and changing attitudes to violence. The study by Kageyama et al. (2020) revealed that a video-based educational program was effective for improving emotional expression, psychological distress, and family empowerment.

We conclude that the violence against parents caused by adult children dispels the myth that in the context of family violence, there is only intimate partner violence and child abuse or that the perpetrators of violence are only partners and parents. Moreover, the literature review revealed issues concerning intervention in cases of violence against parents by adult children (Aas, 2018).

The literature review shows a lack of definitions of violence against parents by their adult children and theories that explain such cases. The critical findings of the systematic literature review of empirical studies on violence against parents by adult children are summarized in Table 2.

**Table 2.** Critical Findings.

Findings	Description
Definition	Only two definitions of violence against parents by adult children. In other literature, there is a general lack of definition(s) of violence against parents by adult children.
Theoretical models	Most of the researched literature does not provide theories explaining violence against parents by adult children. No researcher has developed a model. Some literature includes ideas that come from elder abuse and may coincide with violence against parents by adult children.
The prevalence and characteristics of violence against parents by adult children	The prevalence of violence against parents by their adult children ranges from 5.9% to 40%. The research estimated psychological, physical, and sexual violence and property crime. The victims are on average 70.8–76.5 years old and mostly female. Social isolation and the health status of victims can predict their victimization. Perpetrators are on average 39.6–44.1 years old, not married, can have problems with (mental) health, illegal drug, and alcohol abuse, financial difficulties, and often live together with their parents.
Parents’ responses to and experiences of violence by adult children	Parents face barriers to reporting their own victimization (e.g., internal conflicts, shame) and different dynamics in their relationships with children can develop (e.g., bitter and deprived parenthood, martyrdom).
Social perceptions of (older) parent abuse	Society perceives physical violence against parents and neglect as the most extreme forms of violence, and psychological violence as moderate and mild violence.
Consequences and prevention of violence against parents by adult children	Parental victimization is associated with the risk of post-traumatic stress syndrome. Two programs (Child- and Parent-focused Violence Program, and a video-based educational program) show promising results for reducing violence against parents by adult children.

### Implications for Practice, Policy, and Research

Despite the lack of literature on violence against parents by adult children, the literature review findings are helpful because they show possibilities for designing programs and other activities useful for prevention, detection, and further research. The educational curricula and training of social and health workers, police officers, state prosecutors, and judges could include content on the characteristics and obstacles that may occur while detecting such cases (see, e.g., Aas, 2018). Given that victims often refuse to talk about violence, it seems useful to develop a special approach to encourage victims to speak about violence and report abuse. Two pilot studies show promising findings in prevention that could be harnessed to create preventive programs (Kageyama et al., 2020; Sun & Hsu, 2016).

Although only a few studies provide findings about perpetrators’ employment status and co-residence (Greenberg et al., 1990; Johnson et al., 2022; Lee et al., 2022; von Heydrich et al., 2012), from a policy perspective, it is necessary to research these issues and, in line with the findings, address it via social policy.

In the field of research on violence against parents by adult children, it is necessary to introduce definitions and create a theoretical model to explain the dynamics and risk factors of this type of violence in such relationships. Further research is needed to provide findings about: (a) the characteristics of perpetrators (e.g., the age of perpetrators, their marital and employment status); (b) characteristics of the parent–child relationship (e.g., effects of co-residence and conflicts that occur while living in the same household); and (c) the characteristics of the victims (e.g., substance abuse and health issues). Additional research is called for to investigate the consequences and test preventive approaches, such as the Child and Parent-focused Violence intervention program (Sun & Hsu, 2016) and a video-based educational program (Kageyama et al., 2020). Research that compares current findings on violence against parents by adult children and other types of elder abuse is also called for because it can provide valuable insights into the prevalence, patterns, and impact of different forms of abuse among the elderly population. A valuable source of research may be police crime statistics, documentation from social work centers, and police, prosecutors, and court files. There is a need for specific victimological, especially longitudinal, research to provide insights into the longer progress of these types of violence. Similarly to measuring the prevalence, researchers relied on different research approaches to determine the characteristics of victims and perpetrators. As this makes it difficult to compare the results of various studies, it is a reasonable next step to develop uniform tools to measure the prevalence and identify characteristics. Research should also be conducted on representative samples and with parents and adult violent children since this would enable the generalization of findings to the population, and more detailed insights into the issue from the perspective of perpetrators and victims would be obtained. Such studies showing a significant gap in understanding of this issue have yet to be carried. Research along those lines may yield valuable insights able to inform more targeted interventions and support systems.

The diversity of studies is evident as they have been conducted across a variety of countries (e.g., Dong et al., 2017; Johnson et al., 2022; Kageyama, Solomon, Yokoyama, 2016; Klun et al., 2022). In addition, diversity is included in measured variables related to the race, income, education level, employment status, and marital status of adult children and parents (e.g., Anetzberger et al., 1994; Dong & Li, 2015; Greenberg et al., 1990; Johnson et al., 2018, 2022; Lee et al., 2022). Although these characteristics are often included as part of the demographic data of the samples, future research must delve deeper by analyzing the impact of these factors on the prevalence and dynamics of violence against parents by adult children. Another aspect of the perception of violence is the question of the perception of victimizing parents in institutional environments, such as retirement homes. How parents cope with violence when their children are not dependent on them and what are the myths and stereotypes in this field could also be investigated. The latter could explain why there is social resistance, why nonreporting happens, and why the sexual abuse of parents is rarely mentioned. Findings would be helpful for prevention, detection, and further research. The implications of the systematic literature review for practice, police, and research are summarized in Table 3.

**Table 3.** Implications Held by the Review for Practice, Policy, and Research.

Recommendation	Details
Practice	Designing programs or other activities for health and social services, police, and retirement homes could help detect and prevent violence against parents by adult children
Policy	Employment and housing opportunities for adult children
Research	Conducting longitudinal studies, victimological studies, and studies on representative samples and studies involving both victims and perpetrators

### Limitations

This systematic literature review has several limitations. First, the studies included in the analysis were based on the inclusion criteria, and thus, we did not analyze literature in languages other than English. In addition, we did not exclude studies based on methodological quality (e.g., sample size, sampling). A further limitation is the topic under which the analyzed study is addressed in the context of elder abuse by some authors (e.g., Anetzberger et al., 1994), which made the literature review difficult, especially in the phase of reviewing databases based on titles, keywords, and other information. Apart from the above limitations, there is information lacking as to the age range of the adult children included in the analyzed research, and a lack of definitions of violence against parents by adult children, which might indicate a lack of understanding of the issue. Defining only violence or elder abuse is insufficient since it represents only part of the definition of violence against parents by adult children and does not encompass its key characteristics. Such definitions are too general and do not cover the characteristics of the perpetrators, victims, and the dynamics of the relationship between them. One of the limitations is thus that there may be divergence in the ways different researchers understand the concept of violence against parents by adult children.

## Conclusion

The prevalence of various types of violence against parents by adult children ranges from 5.9% to 40%. The victims are on average 70.8 to 76.5 years old. Their social isolation and health status can predict their victimization. Different dynamics can emerge between a parent and a child in a violent relationship, and victims perceive their victimization in a variety of ways. The essence of such relationships lies in the fluctuating strength and intensity of the violence. They perceive their role in a range of ways and, despite their efforts to stop the violence, the ultimate solution seems to be separation from the parents and the child embarking on an independent life. Violence against (elderly) parents is perceived on different levels (extreme, moderate, and mild violence) of violence. Physical violence and neglect are commonly perceived as extreme forms of violence and psychological violence as moderate and mild violence. Society refuses to think about such violence and does not think about it as fast as other types of family violence.

The literature review raises the question of how such cases are detected, which are the consequences, what is the treatment of victims, and how to prevent such violence. Research on violence against parents by their adult children is lacking, and analyzed studies were largely conducted on nonrepresentative samples, and their findings cannot be generalized to the population. Researchers also used different research approaches, making it challenging to compare the results. The findings thus provide insight into the topic and may be viewed as starting points for further research. Additional research is needed to better understand and strengthen the research findings about the prevalence, characteristics, parents’ responses, and experience of the violence, the social perspective on parent abuse, the consequences, and ways to intervene in and prevent such violence.

## Supplemental Material

sj-docx-1-tva-10.1177_15248380241280955 – Supplemental material for Violence Against Parents by Adult Children: A Systematic Literature Review of Empirical Studies Supplemental material, sj-docx-1-tva-10.1177_15248380241280955 for Violence Against Parents by Adult Children: A Systematic Literature Review of Empirical Studies by Monika Klun, Aleš Bučar Ručman and Danijela Frangež in Trauma, Violence, & Abuse

## Notes

1. The data were taken from the study “Japanese Family Violence and Mental Illness” (Kageyama et al., 2015).

2. The data were taken from the study “Survey on Adverse Experiences of Korean Families with Children” (Ryu et al., 2017, cited in Lee et al., 2022).

3. Data collected in the longitudinal study “MIDUS 2” (Ryff et al., 2006) were used.
