# Supplementary material for: Violence Against Parents by Adult Children: A Systematic Literature Review of Empirical Studies
Source: Trauma Violence Abuse. 2024 Sep 25;26(3):451–67. doi: 10.1177/15248380241280955 (PMC12145486; doi:10.1177/15248380241280955)
Supplement: sj-docx-1-tva-10.1177_15248380241280955 – Supplemental material for Violence Against Parents by Adult Children: A Systematic Literature Review of Empirical Studies [file sj-docx-1-tva-10.1177_15248380241280955.docx]

**Appendix A. Key authors and the snowball method**

Key authors were identified as those who had published three or more contributions. The key authors are Tova Band-Winterstein, Hila Avieli, Masako Kageyama, Yael Smeloy, Georgia J. Anetzberger, Craig Austin, Maria D. Daskalopoulos, Jill E. Korbin, and Phyllis Solomon. Based on this, articles by these authors were determined to be key articles (15 articles) on whose basis a literature review was carried out using the snowball method according to Wohlin (2014). In more detail, backward snowballing was used, which means additional articles were identified using the reference list of the articles. The reference list was reviewed based on all the details included, especially the titles. The inclusion criterion was empirical literature addressing violence against parents by adult children published in English. Literature not meeting the criterion and already found during the systematic literature review was excluded. Literature that met the inclusion criterion was reviewed by abstracts and texts to decide on inclusion. We found one relevant book entitled *The etiology of elder abuse by adult offspring* by G. J. Anetzberger from 1987. Since it was unavailable, the doctoral dissertation by the same author (Anetzberger, 1986) with the title *The etiology of elder abuse by adult offspring: An exploratory study* was analyzed. Further, the literature was reviewed by key authors in the Web of Science and PubMed databases. Similarly, like before, the literature was first reviewed by the titles. The inclusion criterion was empirical literature addressing violence against parents by adult children published in English. The literature that did not fulfill the criterion and already found by the systematic literature review and the snowball method were excluded. Before making the final decision on inclusion, the abstracts and texts of the literature were reviewed. This led us to detect another relevant article (Kageyama et al., 2016b). The process of reviewing literature by key authors and the snowball method was documented in Word files.
